# Gonorrhœal Rheumatism*Read before the Buffalo Medical Club.

**Published:** 1881-06

**Authors:** Herman Mynter


### GONORRHŒAL RHEUMATISM.*

BY HERMAN MYNTER, M. D.

I HAVE in the last year seen several cases of what is generally called gonorrhœal rheumatism, and as the treatment adopted, although differing from the one generally recommended, has been eminently successful, and besides has proven, to my own satisfaction, the local nature of this troublesome affection, I take the liberty to report these cases to you to-night. I readily admit that three or four cases are not sufficient from which to deduce final conclusions; but if the same pathological changes are found in them all, the conclusions are entitled to some weight.

1. Mr. B., 19 years of age, consulted me about a year ago. Six weeks previously he had contracted a gonorrhœa, which was still discharging freely. Three days before I saw him, he had a chill, followed with fever (temperature 103), and increasing pain and swelling of both knee-joints. During the following four days he had high fever, and the exudation in the joints increased to such a degree, and was attended with so much pain, that I considered puncture necessary. About 100 grams of a yellowish, highly fibrinous fluid, in which the microscope revealed innumerable pus-corpuscles, were removed from each joint. The temperature fell immediately to normal, not rising again. The joints were strapped with adhesive plaster and covered with an ice bladder for a few days, after which time, as still considerable tenderness remained, a vesicatory was applied over both knees and the wounds kept open for a week with unguentum sabinæ. Martin's elastic bandage was then applied, and in the course of two weeks the patient was discharged cured.

2. Mr. D., 43 years of age, had two attacks of gonorrhœa, one ten and one five years ago, which on both occasions were followed by inflammation of the left knee-joint.

*Read before the Buffalo Medical Club.

This was so severe that he was confined to his bed for six weeks, and for respectively ten and eight months afterwards had a stiff and swollen knee, and was obliged to walk with crutches. In August, 1880, he contracted a gonorrhœa, which was followed fourteen days afterwards with pain and considerable exudation of left knee, and slight pain and swelling of right tarsus. He had continuous fever, the temperature being about 102, with slight morning remissions. As the pains in the left knee increased to be almost intolerable, and the fever continued in spite of anti-rheumatical treatment, the joint was aspirated on the fifth day, and 200 grams greenish, yellowish exudation, consisting largely of pus, removed. Martin's bandage was applied and an ice bladder put over the joint. The fever disappeared immediately, the exudation did not return, and six days after the puncture the patient was able to leave his bed and walk without any pain.

3. The following case, which has taken a much more serious course, shows, in my opinion, still more clearly the effect of a local treatment and the utter failure of an anti-rheumatic medication. The patient, a man of 52 years of age, contracted a gonorrhœa 14 days previously; August 22d, 1880, he had a severe chill, followed with fever (temperature 103), perspiration and slight pain and exudation in left knee joint, which increased during the following five days. The fever continued high in spite of quinine (centigrammes 50, three times a day) and salicylate of soda (grams 1, four to six times a day). August 27th the joint was aspirated, as the pain was unbearable and could not be relieved except by anodynes, in very large doses, and 150 grams of a fibrinous yellowish fluid was removed, which, under the microscope, showed itself to be clear pus.

Martin's bandage, posterior splint and ice-bladder, were applied. The temperature fell immediately to normal and kept low for two days, when it again commenced to rise, while at the same time the exudation returned in the joint and the pains increased. A large vesicatory was applied over the joint, by which the pains were in some measure mitigated, but as the fever again raised to 103 and no pressure could be tolerated, the joint was again aspirated on September 1, and about 60 grams fluid of similar nature removed. Again the temperature fell temporarily, but the indications returning September 5th, the joint was again aspirated and 50 grams fluid removed, which was of thinner consistency, less yellowish and contained fewer pus-corpuscles.

During the following five weeks the exudation did not return, but there was every evening a slight fever (the temperature being between 101 and 102) while the temperature was normal in the morning, and a diffuse swelling of the whole joint set in with extreme tenderness over the lateral ligaments, especially the internal ligament. Slight movements of the joint were not painful, but jerking it, was extremely so. In spite of the constant use of a posterior splint the tendons commenced to contract, and a slight subluxation and rotation outwards of the tibia commenced, on account of which, extension by weight was used for a time. The limb became considerably straighter, but the extreme tenderness continued. Martin's bandage was again used, but could not be tolerated; sleep could only be produced by large doses of anodynes. Quinine in doses of 25 centigrammes three times a day, tincture of iron in 20-drop doses, and cod liver oil were used, poultices and fomentations with opium were applied continually, but not till in the second week of October did there commence to be a slight change for the better. The fever disappeared, the appetite improved, and the prostration commenced slowly to decrease. At that time there was a slight œdematous swelling around the whole joint and lower part of femur—especially over condyl. internus; and the whole joint was uniformly enlarged as in a white swelling. The tenderness was still so extreme, that the patient was confined to his bed continually and any touch or shaking would produce loud screaming and groaning. October 13th there was still uniform enlargement, especially around condyl. internus, but the pathological condition was in no proportion whatever to the extreme tenderness on the slightest touch or movement. Dr. E. Moore, of Rochester, who had seen the patient in consultation several times, suggested then, that we here might have something similar to an hysterical affection of the joint, or that the knee might be in the state in which a joint may be found after a severe sprain, with subsequent long continued immobility. Passive movements might, therefore, be indicated in spite of the tenderness. The patient was, accordingly, put under the influence of chloroform, the leg freely moved in the joint, and the splint again applied. With some astonishment I found my patient after the operation spending the first comfortable day and night for weeks, while the temperature only rose to 101. During the following two weeks the joint was moved four times under chloroform; every time a slight fever occurred, which lasted 24 hours, and some exudation took place into the joint. Martin's bandage was again applied and tolerated with ease. His general health improved now rapidly, he could sleep without anodynes. his bed-sores healed up, he could bear to have the knee moved without chloroform, and the diffuse swelling of the knee decreased rapidly. On account of the laxity at the joint, he was obliged for half a year to use crutches and a bandage with two lateral steel splints, attached to his shoe, but even this instrument he has now discarded, and a slight looseness of the joint is the only mark the severe disease has left.

4. The etiology of the following case is doubtful, but as the exudation had the same appearance and the treatment was equally successful, I will add it here:

Miss C., 24 years of age, a strong and healthy girl, had for three weeks pain and swelling of left knee without any known cause, loss of appetite, and moderate fever. There was a considerable exudation in the joint, intolerable pain on pressure or movements, slight atrophy of the left calf, and the joint bent about 30°. Four days afterwards, aspiration of the joint was made, by which 150 grams of a fluid was removed of a similar character as that in the three cases mentioned. Martin's elastic bandage, ice bladder and posterior splint was applied. Three days afterward the exudation had nct returned, but there was still considerable pain. A large vesicatory was applied on both sides of patella, and the wounds kept open for eight days. At the end of that time pain and swelling had disappeared, and only considerable stiffness was felt, which disappeared by passive movements and the use of Martin's elastic bandage, in a few weeks.

I know very well that I cannot count this case with certainty, particularly as gonorrhœal rheumatism is seldom found in females, and as the circumstances prevented me from ascertaining whether she had had any urethral discharge. On the other hand, I had here a case of inflammation of the left knee, coming on without any known cause, and on puncture, the exudation had the same characteristics as in the other cases, and the treatment was equally successful.

I shall not try in this paper to give a perfect description of gonorrhœal rheumatism, a disease which is well known to all, (especially through Bumstead's excellent treatise), but simply to make a few deductions from my few cases.

Nobody doubts nowadays that there is a close relation between gonorrhœa and the affection of the joints; and this affection, as Bumstead says, presents certain peculiarities, which in general are sufficient to distinguish it from inflammatory rheumatism. Among these peculiarities, it may be mentioned that it recurs with every attack of a gonorrhœa in persons who once have had it; still, it does not seem that it is the gonorrhœa itself which is the cause, but simply the urethritis, as it may follow a urethritis brought on by other means than the gonorrhœal virus. Here we find the cause why women are so rarely attacked, as they seldom have urethritis; and vulvitis or vaginitis does not produce gonorrhœal rheumatism. Another point is the predilection for the knee-joint, especially the left, and that the disease does not attack a great many joints or change from one to another. That the tunics of the eye, the sciatic nerve, the bursæ, and sometimes the sheaths of the muscles, may be attacked, is well known, and may have occasioned the name gonorrhœal rheumatism.

In regard to the pathological anatomy, Bumstead says "that gonorrhœal rheumatism is essentially an hydarthrosis, and that suppuration very rarely occurs." Other recent authors say the same. This statement does not agree with my limited experience; but as I have punctured all my cases, and examined the fluid with the microscope, while Bumstead only mentions puncture as having been performed by Dieulafoix, my statement ought to have some weight. I found the fluid in all cases to contain a large number of pus-corpuscles, and consequently the disease not to be hydarthrosis, but essentially a suppurative synovitis, in short a local disease. Furthermore, the type of the fever we find speaks for this. In all my cases the fever was considerable before the puncture, and the temperature fell to the normal or almost normal limit immediately after the puncture, and increased first when the exudation recurred, again to disappear by puncture. It was evident to me that the fever was in direct proportion to the severity of the inflammation of the joint and to the amount of matter contained in the fluid.

Whether we now call this fever an inflammatory or a slight pyæmic fever, due to absorption of pus, seems of less importance to me. The connection between the disease of the joints and the urethritis is at present entirely unknown; but I do not see why it might not be of a metastatic nature, as an orchitis following mumps.

The prognosis is, generally speaking, good, in so far as most patients ultimately recover; but the disease has on the other hand an inclination to take a subacute or chronic course and disable the patient for many months. A sufficient number of cases are on record in which the disease, in spite of all treatment, progressed to destruction of the joint, with the formation of abscesses; to make the prognosis grave, especially in weak and debilitated subjects. Bumstead says that recovery can rarely be expected in less than a month or six weeks, and is often delayed for several months or even years.

In regard to treatment, the same rules which may be applied in inflammation of joints from other causes, hold good here. An anti-rheumatical medication has in my hands proven absolutely useless. A local treatment is the all-important one, and only iron and quinine as tonics and antifebrilia, opium to produce sleep, cod-liver oil and good nourishment, are necessary. During the first acute stage, absolute rest must be secured to the joint by aid of a splint; this is so much more necessary, as the disease has a tendency to take a chronic course, and it is absolutely impossible without a splint to prevent the contraction of the muscles and consequently the bending and subluxation of the joint, even if only a moderate exudation occurs; as the knee can contain more fluid when bent than when straight. Ice bladders may be applied over the whole joint, and their effect is grateful to the patient.

If an exudation occurs, it ought to be removed, and if my belief that the disease is a suppurative synovitis, and not a simple hydarthrus, should prove correct by the experience of others, then the earlier we puncture, the better. The puncture acts directly antiphlogistically; the fever falls immediately, and the violent pains disappear. With a fine trocar and antiseptic precautions, no danger can arise from the puncture. It may even be advisable to combine the puncture with a cleaning out of the joint with carbolized water; or, under certain circumstances, to make free incisions in the joint, which, if done antiseptically, need not destroy its motion.

After the puncture Martin's elastic bandage may be applied with good result, and after some days, when the inflammation goes down, a large vesicatory may be used.

Passive movements may be begun early, combined with massage of the joint. These movements are necessarily very painful and may have to be done under chloroform for a time. If the disease should progress and the cartilages and ligaments be affected, the treatment will be the same as in inflammation from other causes, extension, incisions, resection, amputation, etc., as the case in course of time may demand.

Martin's bandage is unbearable as long as the exudation is increasing and the inflammation acute, except after the puncture, but during the subsidence of the disease, beneficial, and its use pleasant, especially combined with rubbing of the joint. To make it easier for the patient to remove and reapply it again, it may, with advantage be used in the form of a many-tailed bandage, (Scultetus bandage). My third case shows, how a joint, after the acute inflammation has subsided, can enter into a state of extreme sensitiveness, possibly from not using it. It is difficult or impossible to decide, when the inflammatory process stops and the nervous element commences. Under such circumstances passive motions may be still more indicated, and act as directly curative, although their use is not without danger and the inflammation may be rekindled. It is in such cases, that the natural bone-setters get their few brilliant results.

